# Rotavirus Vaccine Workshop Held

**DOI:** 10.3201/eid0201.960115

**Published:** 1996

**Authors:** Roger I. Glass

**Affiliations:** World Health Organization, Geneva Switzerland


For further information on WHO's rapid-response unit, contact

**Philippe Stroot**
Health Communications and Public Relations
World Health Organization
Geneva, Switzerland
Phone: 41-22-791-2535
Fax: 41-22-791-4858

### Rotavirus Vaccine Workshop Held

More than 125 participants from at least 15 countries attended the Fifth Rotavirus Vaccine Workshop at the Centers for Disease Control and Prevention (CDC) in Atlanta, Georgia, October 16-17, 1995.

Rotavirus has emerged as the most important cause of severe diarrhea in children worldwide. It is a problem not only in developing countries, where it kills an estimated 870,000 children each year, but also in the United States, where it remains the most important single cause of hospitalization or clinic visits for childhood diarrhea.

Moreover, although studies from many countries indicate that only four serotypes are predominant worldwide, some strains at every site studied cannot be serotyped. In some countries such as India, the diversity of strains is extensive. Further studies are needed to define the extent of cross-protection against these strains that is induced by the vaccine to determine whether additional antigens need to be included in vaccines for such areas.

This workshop included sessions on epidemiology, virology, pathogenesis and immunity, and vaccines currently being tested. Each session had numerous presentations by leaders in the field of rotavirus research. Researchers reported that several live oral rotavirus vaccines, based on animal strains of rotavirus combined with reassortant strains, have been tested in field trials in children. These appear to protect American children against rotavirus and are more efficacious against severe disease. These vaccines like natural protection, are not 100% protective so many investigators are exploring alternative approaches to vaccines such as the use of virus-like particles, native DNA, and microencapsulation of antigens.

No published volume of proceedings from the workshop is planned, but a supplemental issue of the *Journal of Infectious Diseases* scheduled for early 1996 will contain papers from the meeting.

The workshop was held under the auspices of the National Institutes of Health, Emory University School of Medicine, and the World Health Organization.

For further information, contact

**Roger I. Glass**
Global Program for Vaccines and Immunizations
World Health Organization
Geneva, Switzerland
Phone: 41-22-791-2698/2681
Fax: 41-22-791-4860

### International Conference Addresses Preparedness for Emerging Strains of Pandemic Influenza

An international meeting on pertinent issues related to recognizing, identifying, and controlling newly emerging strains of pandemic influenza was held in Bethesda, Maryland, December 11–13, 1995. The conference, "Pandemic Influenza: Confronting a Reemergent Threat," was sponsored by the National Institutes of Health, the University of Michigan, the Centers for Disease Control and Prevention, the Food and Drug Administration, the U.S.-Japan Cooperative Medical Science Program, and the World Health Organization.

Epidemic strains of influenza cause infections almost every year throughout the world because of continuous minor genetic changes in the virus. However, periodically a major change occurs, such as reassortment between mammalian and avian strains of the virus. These pandemic strains are novel to the human immune system and, therefore, can cause substantial disease worldwide. The conference concentrated on issues that would be crucial to controlling an influenza pandemic.

Plenary and workshop sessions examined the following topics: Can pandemics be predicted? What are the specific approaches for pandemic control? What are the advantages and limitations of vaccines and antiviral agents? The workshops also focused on factors contributing to the emergence of pandemic strains and various aspects of surveillance, such as the adequacy of current global surveillance structure for early identification of a pandemic strain, the use of virologic and

More than 125 participants from at least 15 countries attended the Fifth Rotavirus Vaccine Workshop at the Centers for Disease Control and Prevention (CDC) in Atlanta, Georgia, October 16-17, 1995.

Rotavirus has emerged as the most important cause of severe diarrhea in children worldwide. It is a problem not only in developing countries, where it kills an estimated 870,000 children each year, but also in the United States, where it remains the most important single cause of hospitalization or clinic visits for childhood diarrhea.

Moreover, although studies from many countries indicate that only four serotypes are predominant worldwide, some strains at every site studied cannot be serotyped. In some countries such as India, the diversity of strains is extensive. Further studies are needed to define the extent of cross-protection against these strains that is induced by the vaccine to determine whether additional antigens need to be included in vaccines for such areas.

This workshop included sessions on epidemiology, virology, pathogenesis and immunity, and vaccines currently being tested. Each session had numerous presentations by leaders in the field of rotavirus research. Researchers reported that several live oral rotavirus vaccines, based on animal strains of rotavirus combined with reassortant strains, have been tested in field trials in children. These appear to protect American children against rotavirus and are more efficacious against severe disease. These vaccines like natural protection, are not 100% protective so many investigators are exploring alternative approaches to vaccines such as the use of virus-like particles, native DNA, and microencapsulation of antigens.

No published volume of proceedings from the workshop is planned, but a supplemental issue of the *Journal of Infectious Diseases* scheduled for early 1996 will contain papers from the meeting.

The workshop was held under the auspices of the National Institutes of Health, Emory University School of Medicine, and the World Health Organization.

For further information, contact

Roger I. Glass, Global Program for Vaccines and Immunizations, World Health Organization, Geneva, Switzerland; Phone: 41-22-791-2698/2681; Fax: 41-22-791-4860

